# Aqueous sodium borohydride induced thermally stable porous zirconium oxide for quick removal of lead ions

**DOI:** 10.1038/srep23175

**Published:** 2016-03-16

**Authors:** Nadiya B. Nayak, Bibhuti B. Nayak

**Affiliations:** 1 Department of Ceramic Engineering, National Institute of Technology Rourkela, Odisha, 769 008, India

## Abstract

Aqueous sodium borohydride (NaBH_4_) is well known for its reducing property and well-established for the development of metal nanoparticles through reduction method. In contrary, this research paper discloses the importance of aqueous NaBH_4_ as a precipitating agent towards development of porous zirconium oxide. The boron species present in aqueous NaBH_4_ play an active role during gelation as well as phase separated out in the form of boron complex during precipitation, which helps to form boron free zirconium hydroxide [Zr(OH)_4_] in the as-synthesized condition. Evolved *in-situ* hydrogen (H_2_) gas-bubbles also play an important role to develop as-synthesized loose zirconium hydroxide and the presence of intra-particle voids in the loose zirconium hydroxide help to develop porous zirconium oxide during calcination process. Without any surface modification, this porous zirconium oxide quickly adsorbs almost hundred percentages of toxic lead ions from water solution within 15 minutes at normal pH condition. Adsorption kinetic models suggest that the adsorption process was surface reaction controlled chemisorption. Quick adsorption was governed by surface diffusion process and the adsorption kinetic was limited by pore diffusion. Five cycles of adsorption-desorption result suggests that the porous zirconium oxide can be reused efficiently for removal of Pb (II) ions from aqueous solution.

The presence of nanoscale pores in metal oxide such as zirconium oxide nanoparticles play a vital role to enhance the physical and chemical reactivity of nanoparticles and promising potential applications in adsorption, catalysis, gas purification, sensors as well as in biological application^1^,^2^,^3^. But, development of nanopores in zirconium oxide is a great challenge and numerous strategies have been devoted to synthesize nanoscale porous zirconium oxide^4^,^5^,^6^,^7^,^8^. Several synthesis approaches such as sol-gel^9^, precipitation^6^, tape casting^10^, oil emulsion method^11^ and solvothermal^12^ have been employed to develop porous zirconium oxide by using hard or soft templates as well as self-assembly process with the help of specific surfactants to meet the requirement for different applications^5^,^13^. The approach using either templates or self-assembly process using surfactants (such as sodium dodecyl sulfate, ammonium lauryl sulfate, sodium lauryl sulfate) for developing porous materials needs complicated pre-preparation and or post-treating processes. For template-based method, building a porous framework, inset of materials into the template and removing the pore-forming framework is a big challenge^14^,^15^. In addition, self-assembly process is a comparatively simple but removal of surfactants at the end of the fabrication is also a big problem. In these above syntheses, development of porous in the as-synthesized condition as well as sustaining the porous nature up to moderate temperature is also difficult^5^. In addition, zirconium oxide in the form of tetragonal (t) at room temperature was considered to be an important ceramic material and the above synthesis methods have been employed for the development of porous structure. However, poor structural stability (i.e. phase transformation of zirconium oxide from tetragonal to monoclinic (m), when the sample was cooled down from high or moderate temperature to ambient condition) and difficult in removal of template led to affect the potential application of porous zirconium oxide^14^,^16^. Further, in order to develop a porous structure in nanoparticles, it is necessary to form loose or agglomeration free powders in the as-synthesized condition. But it is too difficult to prepare agglomeration free powders through the wet-chemical route without adding any surfactants or templates, because the highly energetic nuclei particles in solution coagulate with each other to form large agglomerate particles by decreasing their surface energy. So, in this context, a new progress has been made in tailoring the synthesis of agglomerated free or loose particles in the as-synthesized condition through gas-bubbles template mechanism. In this method, the gas-bubbles create numerous nucleation sites throughout the solution during synthesis and help to control the agglomeration of nuclei and thus control the size of the particle in the as-synthesized condition. The agglomeration free smaller size particle may form stable tetragonal zirconium oxide up to moderate temperature^6^,^17^. In the same time, the as-synthesized loose particles may develop moderate temperature stable porous particle during calcination process due to coarsening of particles as well as coalescence of voids^18^,^19^,^20^. In gas-bubble template, the gas-bubbles can be generated either by simple blowing a gas into precursor solution or by evolving *in-situ* gas bubbles through chemical reactions^21^,^22^. Gas-bubbles induced porous structure has been emerged as more advantageous than other pore forming methods because of simple room temperature synthesis, clean, template-free, environment friendly and short reaction time^18^,^23^,^24^. Moreover, gases such as N_2,_ Ar, CO_2_, NH_3_ and H_2_S have been used as gas-bubbles template to induce the formation of various porous metal oxides materials^22^,^25^,^26^. In addition to, hydrogen (H_2_) gas-bubbles can also be utilized to develop different types of porous materials^23^,^27^. It was observed that, *in-situ* H_2_ gas-bubbles can be evolved during borohydride synthesis using sodium borohydride (NaBH_4_) as a reagent^28^. Generally, NaBH_4_ is widely identified as a strong reducing power for reducing metal ions to develop metal or metal-boride nanopowders^29^,^30^. But, our group have explored that the borohydride synthesis strategy can not only utilized for producing metal or metal-boride nanopowders but also develop different oxide-based nanomaterials^31^,^32^,^33^,^34^,^35^. However, the detail reaction mechanism on aqueous metal-salts with aqueous NaBH_4_ for producing metal-oxide systems particularly, zirconium oxide is not yet reported. For utilizing the advantage of borohydride concept to other transition metal oxide systems, it is necessary to study the gelation-precipitation reaction mechanism of aqueous NaBH_4_. So, in this paper, we are reporting, a new concept of reaction mechanism between aqueous ZrOCl_2_·8H_2_O and NaBH_4_ with the help of Fourier Transformation Infra-Red (FTIR) spectroscopy. In addition, we are emphasis on advantages of aqueous NaBH_4_ for the development of loose particles in the as-synthesized condition and also development of thermally stable porous zirconium oxide nanomaterials for removal of toxic ions for environmental applications.

## Results and Discussion

**Figure 1:**
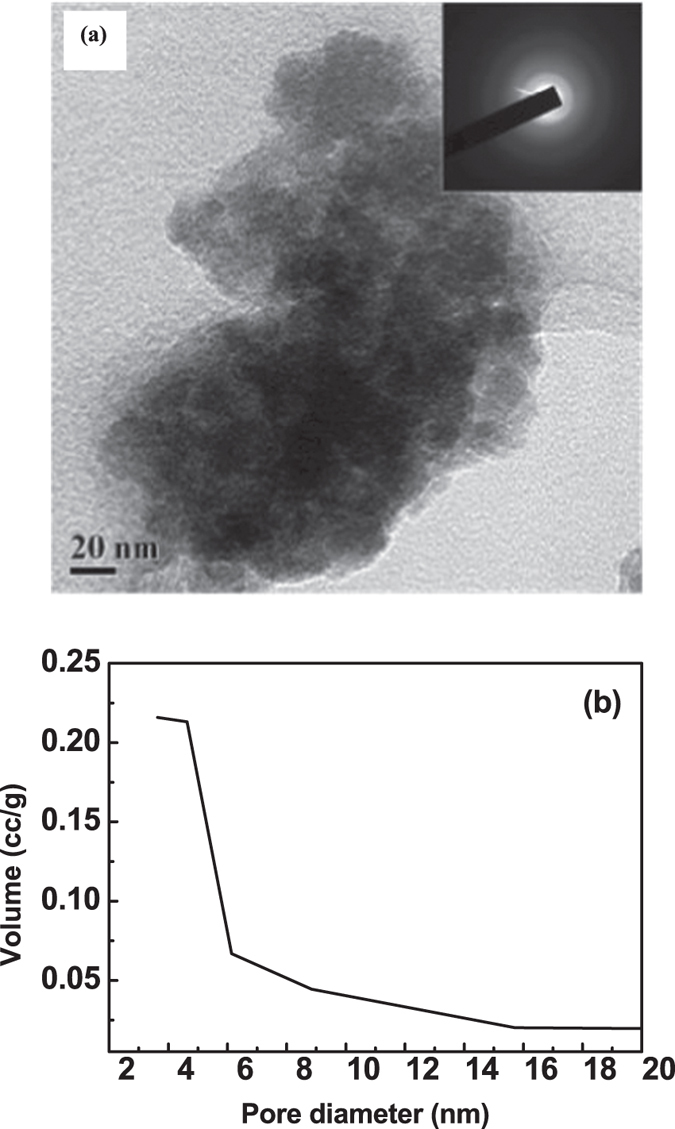
TEM micrograph of borohydride derived as-synthesized zirconium hydroxide (**a**). Hazy electron diffraction pattern in the inset of (**a**) indicates that the powders are amorphous in nature. BJH curve of as-prepared samples is shown in (**b**).

Powder morphology of the borohydride derived as-synthesized zirconium hydroxide powders was studied using Transmission Electron Microscopy (TEM) and was shown in Fig. 1(a). The nature of as-synthesized zirconium hydroxide powder was found to be loose having fine size of ~3–4 nm and was well-separated with each other by voids. The as-synthesized powders was found to be an amorphous in nature, as confirmed from the hazy electron diffraction pattern, indicated in the inset of Fig. 1(a). The BJH curve obtained from BET-isotherm was shown in Fig. 1(b). It indicates that the as-prepared powders exhibit an average pore size of ~4 nm, which was well correlated with TEM result.

So, borohydride based gelation-precipitation reaction is favourable for the formation of loose porous nature of zirconium hydroxide in the as-synthesized condition. Thus, it is justify looking into the insights of the borohydride reaction mechanism between aqueous ZrOCl_2_·8H_2_O and NaBH_4_. The following reaction mechanism was discussed in detail. It is well-known that in aqueous solution, hydrolysis of NaBH_4_ proceeds to form two species such as tetrahydral boron [B(OH)_4_^−^] and hydrogen (H_2_) gas bubbles as per equation (1)^28^




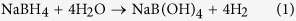




When aqueous NaBH_4_ (initial pH ~11) associated with two active species such as B(OH)_4_^−^ and H_2_ gas-bubbles was added to the aqueous solution of ZrOCl_2_·8H_2_O (initial pH ~0.3), zirconium hydroxide [Zr(OH)_4_] nuclei are start to grow via hydroxide ion exchange reactions along with the formation of trigonal boron [B(OH)_3_] and H_2_ gas-bubbles as per equation (2). The B(OH)_3_ so formed in aqueous medium converts to tetrahedral boron, as per equation (3).




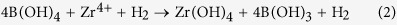







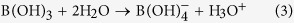




**Figure 2:**
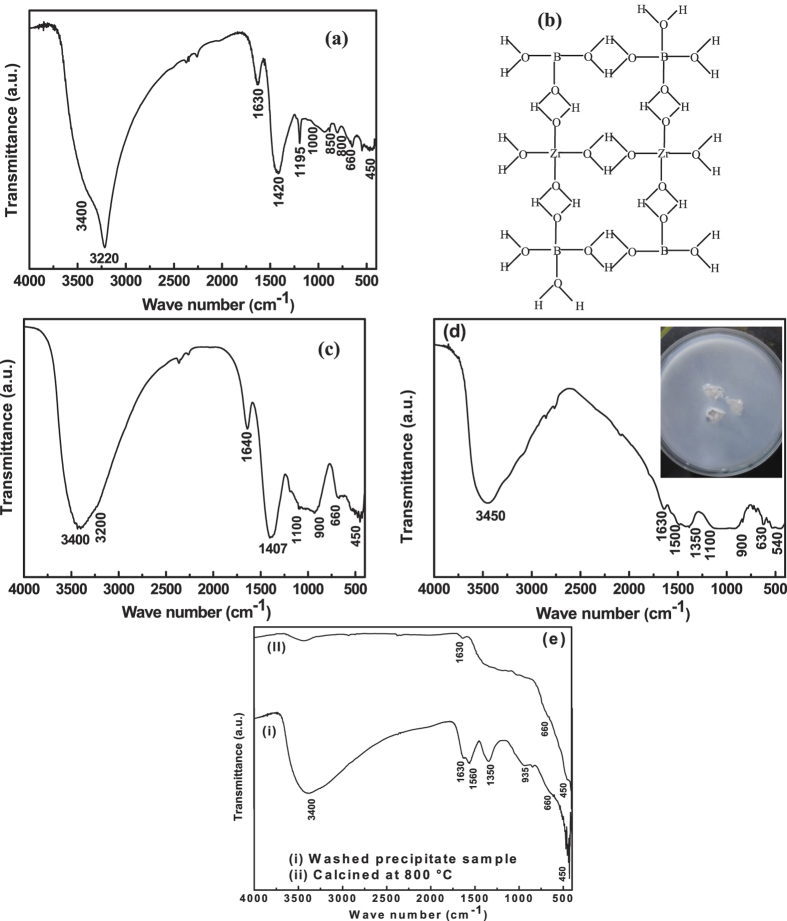
FTIR spectra of un-washed gel sample (**a**), schematically representation three-dimensional network of Zr(OH)_4_ with boron species (**b**), FTIR spectra of un-washed precipitate sample (**c**), FTIR spectra of solid borate sample (**d**), [Solid pieces of boron complex were phase separated from precipitation as shown in inset of (**d**)] and (**e**) FTIR spectra of washed precipitate and calcined (800 °C) sample.

The percentage of B(OH)_3_ and B(OH)_4_^−^ in solution depends on the pH of the precursor solution. At lower pH value, B(OH)_3_ is more dominate than B(OH)_4_^−^, whereas B(OH)_4_^−^ is more dominate at higher pH^36^ During initial stage of synthesis, the highly energetic Zr(OH)_4_ species are formed in presence of trigonal boron and H_2_ gas-bubbles. As the reaction proceeds with continuous addition of aqueous NaBH_4_, pH of the precursor solution increases. At pH ~2.8, a viscos gel-network polymeric chain was observed. Generally, the gelation pH of Zr(OH)_4_ is ~4, while using the common precipitating agent such as NH_4_OH^37^. The sharp decrease of gelation to a pH~2.8, while using aqueous NaBH_4_ indicates that the boron species strongly participates during gelation. To support this gelation mechanism, Fourier Transformation Infra-red spectroscopy (FTIR) of the gel sample (un-washed) was performed and shown in Fig. 2(a). The FTIR peak around 1420 cm^−1^ and 1195 cm^−1^ indicates the stretching and bending vibration of B-OH bond in trigonal boron respectively^38^. Both the stretching and bending vibration of trigonal boron are shifted to a higher frequency in compared to the stretching and bending vibration of B-OH of pure B(OH)_3_ boric acid solution (stretching at 1410 cm^−1^ and bending at 1148 cm^−1^). This shifting is likely due to the strengthening of B-OH bond during gelation with Zr(OH)_4_^39^. In addition, the broad band in the range of 1000 cm^−1^ to 850 cm^−1^ centered at 950 cm^−1^ was assigned to stretching vibration of tetrahedral boron^40^. This broadening nature is due to the formation of intermolecular hydrogen bonding of Zr(OH)_4_ with B(OH)_4_ during gelation process^41^. Thus, FTIR spectra of the gel sample indicate the participation of both boron species during gelation process. The nature of peak in the range of 3000 cm^−1^ to 3500 cm^−1^ is also a strong indicator for the participation of boron species. The narrow sharp band at 3220 cm^−1^ indicates the polymerization via intermolecular hydrogen bonding between Zr(OH)_4_ with boron species. A lower intense peak at 3400 cm^−1^ indicates a minute polymerization among Zr(OH)_4_ units via inter molecular hydrogen bonding^42^. The additional band at 800 cm^−1^ is due to bending vibrations of B–O–B^43^. Furthermore, the band at 660 cm^−1^ and 450 cm^−1^ are assigned to vibrational modes of Zr-O^37^. The peak at 1630 cm^−1^ corresponds to O-H of water. Also during gelation process, the H_2_ gas-bubbles were considered to be trapped within the gel-network^44^.

From the FTIR analysis, it was confirmed that the gel-network polymeric chain was due to the polymeric nature of both Zr(OH)_4_ and boron species^39^. This boron species are able to form inter molecular crosslinking through hydroxyl group with Zr(OH)_4_ nuclei as shown in the schematic diagram of Fig. 2(b).

Again precipitation process was followed with vigorous stirring and further addition of aqueous NaBH_4_. In this process, the pH of the precursor solution gradually increases along with dissociation of gel-network as well as the trapped H_2_ gas-bubbles became active and mobile. The process of precipitation was continued till the pH reaches at 10. To understand the precipitate mechanism, FTIR of the dried un-washed precipitate powders (collected at pH 10) was performed and is shown in Fig. 2(c).

The band at 660 cm^−1^ and 450 cm^−1^ are assigned to vibrational modes of Zr-O. The reduction in FTIR intensity of 3200 cm^−1^ along with increase in FTIR intensity of 3400 cm^−1^ indicates the detachment of boron species from zirconium hydroxide species during gelation to precipitation process. The detached boron species is basically an inter-coordinated boron complex and it was possible through intermolecular hydrogen bonding among boron species only when pH of the solution increases beyond 9^45^. The detachment of boron complex was confirmed from the decrease of stretching and bending vibration of B-OH from 1420 cm^−1^ to 1407 cm^−1^ during gelation to precipitation process. The broad peak starting from 1100 cm^−1^ to 900 cm^−1^ was also a strong indicator for the formation of boron complex^46^. However, the formation of a solid piece of boron complex from the precipitation solution is a slow process. A solid piece of boron complex was phase separated out from the solution when the precipitate solution was kept for two to three days. The solid pieces of boron complex in the precipitated solution were shown in the inset of Fig. 2(d). To understand the band position, FTIR of the solid piece was performed and is shown in Fig. 2(d).

The broad peak at 3450 cm^−1^ was assigned due to stretching mode of O–H band of boron complex. The two major broad peak ranges from 1100 cm^−1^ to 900 cm^−^1 and 1500 cm^−1^ to 1350 cm^−1^ corresponds to vibrational modes of boron complex. These two broad peaks are due to the polymerization of the boron species^41^,^43^. The peaks at about 630 cm^−1^, and 540 cm^−1^ are the characteristic frequencies of symmetric vibration of boron complex^46^. The peak at 1630 cm^−1^ was assigned as the O-H vibrational mode of water.

From above FTIR spectra, it was confirmed that boron species are strongly participated during gelation and these boron species in the form of boron complex were phase separated out during precipitation process. So, the aqueous borohydride process is not only act a reducing agent but also act as a precipitating agent (analogous with NH_4_OH) to produce zirconium hydroxide precipitate powders.

Further to understand the nature of vibrational modes of washed sample, the un-washed precipitate powders were washed several times and dried. The washed and dried as-prepared sample was calcined at 800 °C. The FTIR spectra of both washed as-prepared and calcined (800 °C) zirconium oxide powders are shown in Fig. 2(e). The complete absence of vibrational modes of boron species was observed in the as-prepared as well as calcined samples. The band position at 3400 cm^−1^, 1630 cm^−1^, 1560 cm^−1^ and 1350 cm^−1^ of as-prepared sample are due to O-H vibration modes of H_2_O. The peak position at 450, 660 and 935 cm^−1^ are due to the Zr-O vibration modes^37^.

**Figure 3:**
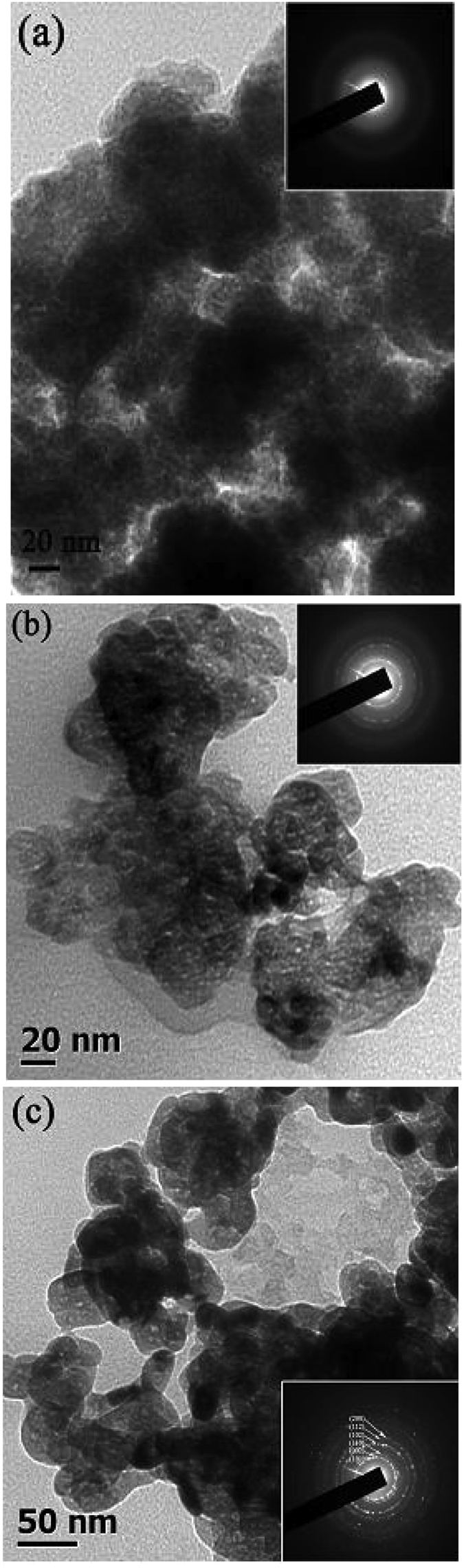
TEM micrographs of as-synthesized powders calcined at (**a**) 400 °C, (**b**) 600 °C and (**c**) 800 °C. Inset of each micrographs show electron diffraction pattern.

To find out the existence of loose nature in zirconium oxide, the as-prepared amorphous zirconium hydroxide powders were thermally heat-treated at different calcination temperatures in the range of 400 °C to 800 °C. Powder morphology were analyzed using TEM. Figure 3(a–c) show TEM micrographs of as-synthesized powders calcined at 400 °C, 600 °C and 800 °C, respectively.

Electron diffraction pattern was also performed on these three samples and are shown in the inset of Fig. 3. Electron diffraction pattern in the inset of Fig. 3(a) indicates that the amorphous nature of zirconium hydroxide remains the same. Comparing the two different types of electron diffraction pattern such as hazy ring in inset of Fig. 3(b) and sharp ring in inset of Fig. 3(c), it was further confirmed that minute amount of amorphous nature still remain along with crystalline nature at 600 °C, but forms purely crystalline at 800 °C. The sharp ring pattern at 800 °C was indexed with the tetragonal form of zirconium oxide.

**Figure 4:**
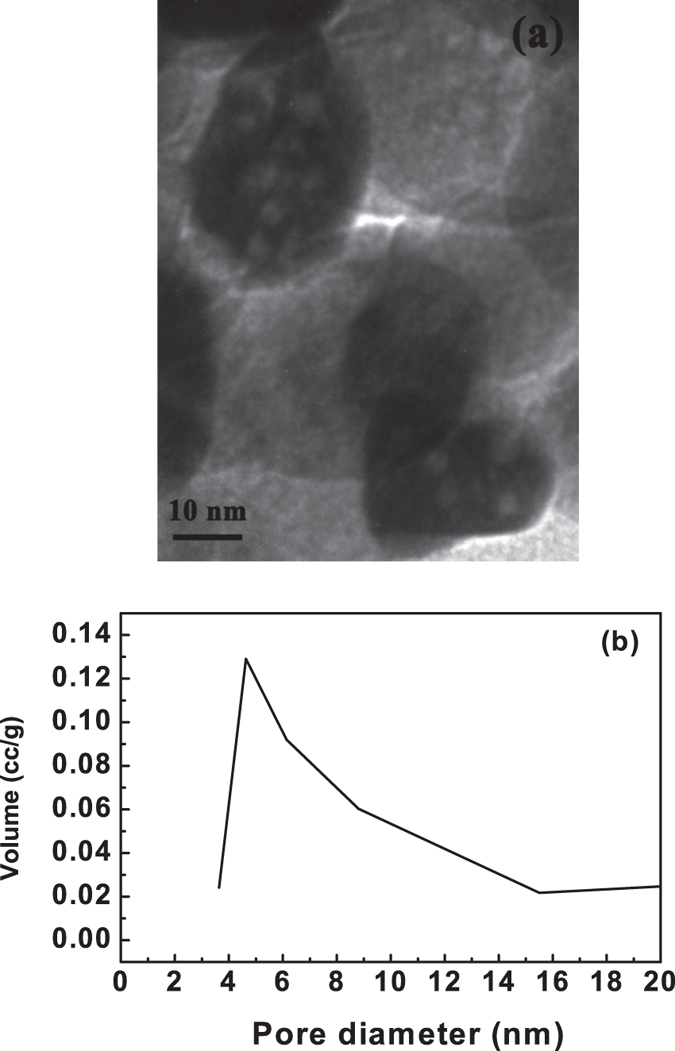
Higher magnified TEM micrograph (**a**) and BJH curve (**b**) of porous zirconium oxide, calcined at 800 °C.

The TEM micrograph of Fig. 3(a) indicates that the loose nature of zirconium hydroxide remains unchanged up to 400 °C. At this temperature, the particles are also well separated with each other by voids with analogues with TEM micrograph (Fig. 1a) of as-synthesized powders. A trapped pore based porous nature of zirconium oxide was observed at 600 °C, as confirmed from Fig. 3(b). The porous nature was also found to be present in the zirconium oxide sample, calcined at 800 °C, as shown in Fig. 3(c). At this temperature, trapped pore and occasional large voids are also observed. However, the particles are agglomerated in nature at this temperature and to visualize one single particle and its porous nature, a higher magnified TEM micrograph of calcined (800 °C) zirconium oxide was represented in Fig. 4(a). It was found that nanoscale pores (~3/4 nm) were well preserved in each individual zirconium oxide nanoparticles, having particle size of ~30 nm. Further, the pore size distribution of calcined (800 °C) zirconium oxide was studied using BJH curve and shown in Fig. 4(b). The BJH curve also indicates that the porous zirconium oxide exhibits wide pore size distribution in the range of 3.6 nm to 15.8 nm, with an average pore diameter of 4.6 nm, which was well correlated with TEM result.

Further, it was necessary to understand the pore evolution mechanism and it was discussed based on the following steps. The first step is the involvement of evolved H_2_ gas-bubbles during the nucleation of Zr(OH)_4_ species to form loose zirconium hydroxide in the as-synthesized condition. During borohydride synthesis, the H_2_ gases generated in the precursor solution were released as gas-bubbles, which act as free-templates^19^,^20^,^22^,^47^. These gas-bubbles create numerous gas–liquid interface aggregation centres throughout the precursor solution during synthesis process^47^. These gas-liquid interface centres help to reduce the interfacial energy of highly energetic Zr(OH)_4_ species through their surface attachment^48^. The surface attached Zr(OH)_4_ nuclei were well separated by gas-bubbles and thus it help to prevent continuous agglomeration of Zr(OH)_4_ species among themselves^22^. The size of Zr(OH)_4_ may be controlled depending on the quantity of gas-bubbles created during synthesis. However, the quantity of evolved gas-bubbles is difficult to quantify. But, in our previous observation^33^, it was confirmed that the quantity of gas-bubbles in the aqueous solution is higher in constant pH method and decreases from precipitate to gelation process. It was also found that the crystallite or particle size of zirconium oxide strongly depends on the way of synthesis and it was due to the participation of different amount of gas-bubbles. In the same time, the participation of different quantity (based on way of synthesis) of gas-bubbles affects the agglomeration of the Zr(OH)_4_ during synthesis. The higher amount of the gas-bubbles, more is the nucleation centres and thus supresses the agglomeration of *Zr(OH)*_*4*_. Whereas lower the amount of gas-bubbles during synthesis led to agglomerate the Zr(OH)_4_ nuclei and finally the cluster size of Zr(OH)_4_ became higher. However, in this present study, the precipitation derived as-synthesized zirconium hydroxide powder prepared via borohydride route was found to be loose in nature and thus it was assumed that sufficient amount of gas-bubbles are participating during precipitation process and help to suppress the agglomeration process of Zr(OH)_4_ nuclei during synthesis. In the solution state, the H_2_ gas-bubbles are surrounded by Zr(OH)_4_ nuclei, but these gas-bubbles create interconnected voids due to escaping of gas-bubbles in dry state. Further, the particles of as-synthesized powder are well separated with each other by voids as seen from Fig. 1(a), and thus assume that there is no presence of trapped hydrogen gas in the dry state.

The second step involves the formation of trapped pore (within one particle) and or large voids (within some agglomerated porous particles) when the loose nature of as-synthesized zirconium hydroxide undergoes calcination process. During initial stage of calcination process i.e. up to 400 °C, it is basically the decomposition of attached crystalline water in Zr(OH)_4_. The uniformity of the as-synthesized amorphous zirconium hydroxide powders help to maintain the original pore structure i.e. loose nature of zirconium hydroxide up to 400 °C. This indicates that the presence of intra-particle voids may inhibit the mass transfer between loose nanoparticles and also help to restrict the coarsening of particles, during calcination up to 400 °C^49^. With increase in calcination temperature up to 600 °C, the seed crystals start to develop within the loose amorphous matrix. These growing nanocrystals impinge in the matrix and thus trapping inter-particulate voids as trapped pore. Again, while heating the sample from up to 800 °C, the coarsening of fine particles along with coalescence of existing pores take place. Occasional large voids can be observed which are formed as a result of excessive necking between particles and coalescence of the adjoining pores.

**Figure 5:**
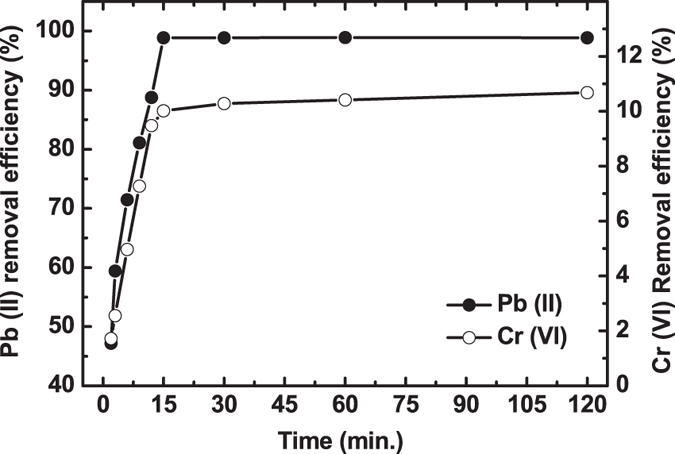
Removal percentage of Cr(VI) and Pb(II) with different interval of time.

The formation of moderate temperature stable porous zirconium oxide obtained from borohydride synthesis is found to be attractive and may find potential applications for the adsorption of heavy metal ions for industrial waste water treatment. In this context, the removal efficiency (in %) of Cr(VI) or Pb(II) at different interval of time was performed and is shown in Fig. 5. The adsorption process by the porous zirconium oxide is time independent and within 15 minutes, the removal efficiency of Cr(VI) and Pb (II) was found to be ~10% and ~99%, respectively. The removal percentage of toxic metal ions by the porous zirconium oxide is selective in nature. So, the borohydride derived porous zirconium oxide may be a strong candidate for almost complete removal of Pb (II) toxic ions from water solution.

**Figure 6:**
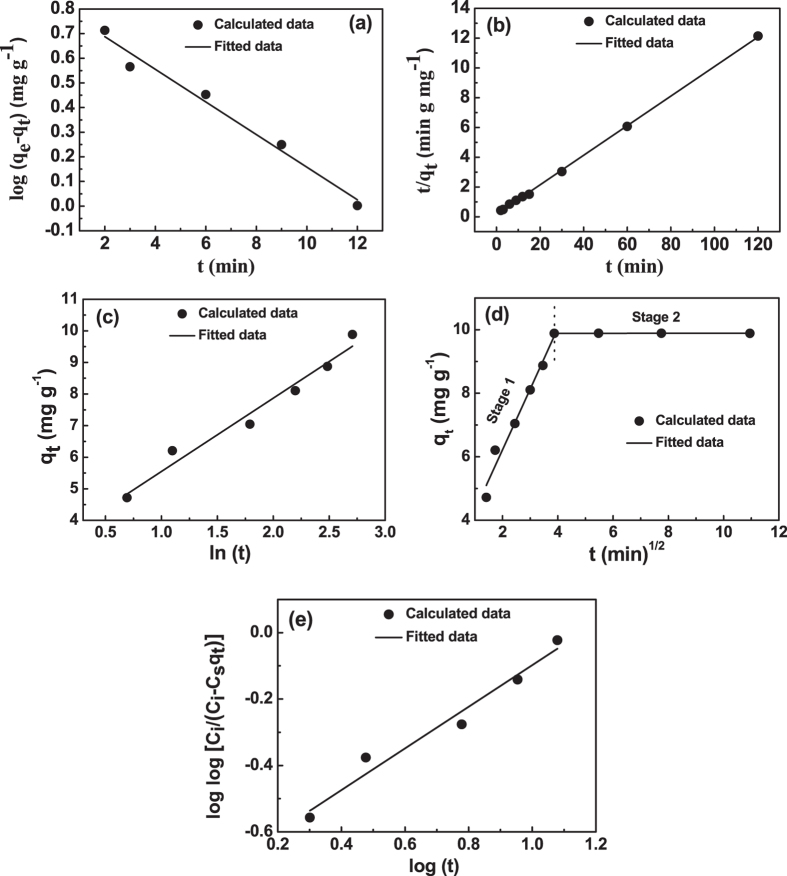
Pseudo-first order (**a**), pseudo-second order (**b**), Elovich (**c**), Intra-particle diffusion (**d**) and Bangham (pore diffusion) (**e**) kinetic plot for adsorption of Pb (II) by porous zirconium oxide powders.

Further, adsorption kinetic mechanism as well as regeneration of Pb (II) loaded zirconium oxide sample was studied. In order to examine the controlling mechanism of the adsorption process, pseudo-first order, pseudo-second order, Elovich, intra-particle diffusion (Weber and Morris’ equation) and Bangham’s (pore diffusion) kinetic models were used. The experimental data were treated with the above five kinetic models and the equations of the different kinetic models are given in supplementary Table S1 online. The kinetic parameters determined for five kinetic models are given in Table 1. The graphs for the pseudo-first order, pseudo-second order, Elovich, intra-particle diffusion (Weber and Morris’ equation) and Bangham’s model (pore diffusion) are shown in Fig. 6(a–e), respectively. The pseudo-first order and pseudo-second order are the most widely used rate equations to describe the adsorption of adsorbate from the liquid phase^50^. The pseudo-first order graph [Fig. 6(a)] was found to be linear with a correlation coefficients of R^2^ = 0.9747, indicating the possible applicability of pseudo first-order model in the present study. But, the pseudo-second order graph [see Fig. 6(b)] was also found to be linear with a correlation co-efficient value [R^2^ = 0.9995] higher than pseudo first-order model. Moreover, the q_e cal_ (mg/g) value determined from pseudo-first order and pseudo-second order equation was found to be 6.605 and 10.07, respectively. The q_e cal_ (mg/g) value obtained from pseudo-second order was very close to the experimental q_e exp_ (mg/g) value (9.88). So, the experimental data to pseudo-first order rate equation suggested the non-applicability of pseudo-first order kinetic in predicting the mechanism of Pb (II) adsorption process. However, higher value of correlation co-efficient (R^2^) and nearly close matching value of calculated and experimental q_e_ value indicates the applicability of pseudo-second order kinetics in this present study. The superior fit of the pseudo-second-order model along with matching of calculated and experimental q_e_ (mg/g) data implies that the adsorption process was surface reaction controlled with chemisorption involving valence forces through sharing or exchange of electrons between adsorbent and adsorbate^50^,^51^. Further, the quick adsorption of Pb (II) by porous zirconium oxide powder was observed after a short time period, which led to examine the experimental data with the Elovich kinetic model and the graph is shown in Fig. 6(c). The Elovich equation assumes the presence of active sites on the adsorbent surface, which led to chemisorption^52^,^53^. Additionally, the correlation co-efficient (R^2^) value for Elovich model was lower than the pseudo-second order, but still rather high. So, Elovich model suggests that chemisorption was the main adsorption controlling mechanism. However, the initial rapid Pb(II) adsorption within 15 minutes also suggests that more than one mechanism may also involve in the process^54^. The pseudo-first order, pseudo-second order and Elovich model can not identify the influence of diffusion on adsorption. So, Weber and Morris’ equation (intra-particle diffusion) and Bangham’s model (pore diffusion) were further analyzed. Rate of adsorption is frequently used to analyze nature of the ‘rate-controlling step’ and the use of the intra-particle diffusion model has been greatly explored. It was evident from the graph of Fig. 6(d) that the first linear portion (Stage I) was attributed to the immediate utilization of the most readily available adsorbing sites on the adsorbent surfaces. The second plateau path (Stage II) indicates very slow diffusion of adsorbate from surface site into the inner pores^55^. Thus, the rapid initial portion of Pb (II) adsorption may be governed by the surface diffusion process and later part is controlled by pore diffusion. The correlation coefficient (R^2^) obtained from Weber and Morris’ equation was found to be 0.97, which is still higher value and also the intercept of the line fails to pass through the origin due to difference in the rate of mass transfer in the initial and final stages of adsorption and indicates some degree of boundary layer control which implies that intra-particle diffusion is not only the rate controlling step^55^,^56^. Further, the experimental data were used to confirm the pore diffusion as one of the rate-controlling steps using Bangham’s equation. The graph obtained [Fig. 6(e)] using Bangham’s equation was found to be linear with a quite good correlation coefficient R^2^ = 0.958 indicating that the contribution of pore diffusion to the overall mechanism of Pb(II) adsorption could not be neglected and may play a role in controlling the rate of adsorption. The adsorption kinetics was pore diffusion controlled and the diffusion into the pores of the adsorbent was not the sole rate-determining process.

**Figure 7:**
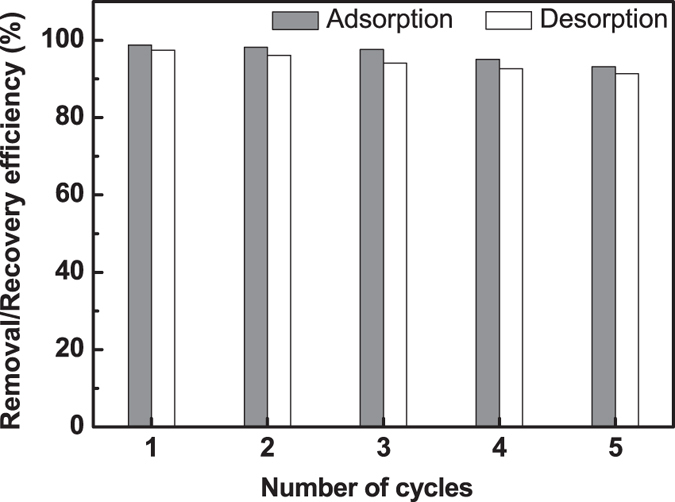
Removal (during adsorption) and recovery (during desorption) percentage of Pb(II) as a function of number of cycles.

When an adsorbent is applied for adsorption of toxic ions, the possibility of regeneration of the adsorbent is of great importance from application point of view. Efficient removal of loaded metal from the adsorbent was necessary to ensure their long term use for repeated adsorption-desorption cycles. The percentage removal efficiency during adsorption and recovery percentage of Pb(II) during desorption for each cycle was determined and shown in Fig. 7. This result indicates that the porous zirconium oxide could be employed on several times for adsorption process without significant losses of its initial capacity of adsorption. It was observed that the removal percentage of Pb(II) was slightly decreases from ~99% to ~93% from the first to the fifth cycle. This may be due to the non-leaching of previously adsorbed Pb(II) ions that resisted to the desorption process. So, the efficient reuse of the Pb(II) loaded zirconium oxide sample was found to be possible and can be applied to the removal of toxic metals from wastewater efficiently.

## Conclusions

For the first time, we are reporting the advantages of aqueous NaBH_4_ for facilitating in gelation and precipitation with aqueous ZrOCl_2_·8H_2_O and producing loose and porous zirconium oxide nanopowders. The boron species such as trigonal and tetrahedral boron are strongly participate in gel-network polymeric chain with Zr(OH)_4_ during gelation process. However, the boron complex in the form of solid pieces was phase separated from the precipitated solution after completion of the precipitation reaction. Additionally, the evolution of *in-situ* H_2_ gas-bubbles creates numerous gas–liquid interface aggregation centres during borohydride synthesis and plays an important role to develop loose zirconium hydroxide powders in the as-synthesized condition. The trapped pore and or large voids are observed, when the loose nature of as-synthesized zirconium hydroxide undergoes calcination process and the porous structure of zirconium oxide was stable up to 800 °C. Without any surface modification the porous zirconium oxide powders were able to adsorb almost all Pb(II) ions within few minutes at normal pH condition. The adsorption mechanism was analyzed using five kinetic models (pseudo-first order, pseudo-second order, Elovich, intra-particle diffusion and Bangham). The superior fitting of pseudo-second order as well as quite good fitting of Elovich model indicates that the main adsorption controlling mechanism was chemisorption. However, the initial rapid Pb(II) adsorption within short period of time involved more than one mechanism. Based on intra-particle diffusion and Bangham’s model, it was further suggests that the quick adsorption was attributed to the immediate utilization of the most readily available adsorbing sites on the adsorbent surfaces and the adsorption kinetics was limited by pore diffusion. Further, quite high efficiency of five cycles of regeneration suggest the importance of porous zirconium oxide for the removal of toxic ions for environmental application.

## Methods

### For powder preparation

Borohydride synthesis via gelation-precipitation method was adopted to synthesize zirconium oxide powders. For preparation of zirconium oxide powders, analytical grade (99.9% pure) reagents of octa-hydrated zirconium oxy-chloride (ZrOCl_2_·8H_2_O) and NaBH_4_ were used as a starting material. Two different aqueous solutions of ZrOCl_2_·8H_2_O and NaBH_4_ were prepared separately. Borohydride reaction was conducted at room temperature with drop wise addition of aqueous NaBH_4_ to a beaker containing aqueous ZrOCl_2_·8H_2_O, with constant stirring using a magnetic stirrer. During synthesis, gelation took place at pH ~2.8 and further pH of the precursor was increased to ~10, with addition of aqueous NaBH_4_ via precipitation process. After completion of the reaction, the precipitate powders were washed, dried and then calcined at different temperatures (400 °C, 600 °C and 800 °C) for 1 h. Powder morphology was studied using Transmission Electron Microscopy (TEM). Pore size distribution was analyzed using Barrett-Joyner-Halenda (BJH) method by considering Brunauer–Emmett–Teller (BET) isotherm behavior.

### For removal efficiency of metal ions with time

To find out the removal efficiency (in %) of Cr(VI) or Pb(II) at different interval of time, two different solutions of potassium chromate (10 ppm) and lead nitrate (10 ppm) were prepared separately using distilled water at normal pH (~7). Calcined (600 °C) porous zirconium oxide of 10 mg was added in 10 ml of the above chromium and lead solutions. These solutions were stirred continuously at room temperature. At different interval of time (2, 3, 6, 9, 12, 15, 30, 60 and 120 minutes), the samples were collected via filtration. The filtered solutions were analyzed using atomic absorption spectroscopy. The removal efficiency (in percentage) of Cr(VI) or Pb(II) at different interval of time was calculated using the formula: [{(C_o_-C_e_)/C_o_}×100], where C_o_ (ppm) and C_e_ (ppm) are the initial and equilibrium concentration of adsorbate in the solution.

### For regeneration of Pb(II) loaded zirconia

In order to check the regenerative capacity of adsorbent, desorption study was carried out using the filtered Pb (II) loaded zirconium oxide sample, collected after 30 minutes of adsorption time (because the adsorption process was more than 98% complete within this time). Desorption was carried out by agitating the Pb(II) loaded zirconium oxide with 5 ml of desorbing agent HNO_3_ (0.5 M) solution. After agitating for 1 hour, it was filtered and the filtrates were analyzed using atomic absorption spectroscopy for determination of recovery percentage of Pb(II) during desorption process. The filtered samples were dried and then again suspended in Pb(II) containing solution for next adsorption run. Five cycles of adsorption–desorption were carried out to examine the capability of the zirconium oxide sample to retain Pb(II) removal capability for regeneration.

## Additional Information

**How to cite this article**: Nayak, N. B. and Nayak, B. B. Aqueous sodium borohydride induced thermally stable porous zirconium oxide for quick removal of lead ions. *Sci. Rep.* **6**, 23175; doi: 10.1038/srep23175 (2016).

## Supplementary Material

Supplementary Information

## Figures and Tables

**Table 1:** Kinetic parameters of Pb (II) using porous zirconium oxide as adsorbent.

Kinetic parameters		
Pseudo-first order model	q_e, exp_ (mg/g)	9.888
	q_e, cal_ (mg/g)	6.605
	K_1_ (min^−1^)	0.1523
	R^2^	0.9747
	Rate equation	y = −0.06617 x + 0.81993
Pseudo-second order model	q_e, cal_ (mg/g)	10.07
	K_2_ (g mg^−1^ min^−1^)	0.0608
	R^2^	0.9995
	Rate equation	y = 0.0993 x + 0.16197
Elovich model	a_e_ (mg g^−1^ min^−1^)	9.319
	b_e_ (g mg^−1^)	0.4308
	R^2^	0.963
	Rate Equation	y = 2.32126 x + 3.22667
Inter-particle diffusion model	K_i_ (mg g^−1^min^−0.5^)	1.91154
	C	2.39608
	R^2^	0.97003
	Rate equation	y = 1.91154 x + 2.39608
Bangham’s model	K_b_ (mL g^−1^ L^−1^)	122.26
	α	0.62723
	R^2^	0.958
	Rate equation	y = 0.62723 x −0.7250
