# Supplementary material for: Aqueous sodium borohydride induced thermally stable porous zirconium oxide for quick removal of lead ions
Source: Sci Rep. 2016 Mar 16;6:23175. doi: 10.1038/srep23175 (PMC4793254; doi:10.1038/srep23175)
Supplement: Supplementary Information [file srep23175-s1.pdf]

Supplementary information

Aqueous sodium borohydride induced thermally stable porous zirconium oxide for quick removal of lead ions

Nadiya B. Nayak, Bibhuti B. Nayak*

Department of Ceramic Engineering, National Institute of Technology Rourkela
Odisha, India 769 008

*Corresponding Author's email: bbnayak@nitrkl.ac.in, bibhutib@gmail.com (Bibhuti B. Nayak)
Phone: +91 661 246 2209 (O)

Supplementary Table S1: The equations of the different kinetic models

Kinetic model	Equation	References
pseudo-first order	$\log(q_e - q_t) = log q_e - \frac{K_1 t}{2.303}$	1
pseudo-second-order	$\frac{t}{q_t} = \frac{1}{K_2 q_e^2} + \frac{t}{q_e}$	2
Elovich	$q_t = \frac{ln\, a_e b_e}{b_e} + \frac{1}{b_e} \ln t$	3
Intra-particle diffusion	$q_t = K_i t^{1/2} + C$	4,5
Bangham's model	$\log log \left[\frac{C_i}{C_i - C_s q_t}\right] = \log \left[\frac{K_b C_s}{2.303V}\right] + \alpha \log t$	6

Nomenclature:

q_e and q_t is the amount of chromium (mg g^{-1}) adsorbed at equilibrium and at time t, respectively.

K_1 (min^{-1}) is the rate constant for pseudo-first order adsorption reaction.

K_2 is the rate constant (g mg^{-1} min^{-1}) for pseudo-second order reaction.

a_e is the initial adsorption rate (mg/g min).

b_e is related to the extent of surface coverage and activation energy for chemisorption (g/mg).

K_i is the intra-particle diffusion rate constant (mg g^{-1} $min^{1/2}$).

C is the intercept.

C_i is the initial concentration (g/L)

C_s is the weight of adsorbent used per liter of solution (g/L).

α and k_b are constants

V is the volume of solute (mL)

Supplementary references

1. Lagergren, S. About the theory of so-called adsorption of soluble substances. *K. Sven. Vetenskapsakad. Handl.* **24**, 1-39 (1898).
2. Ho, Y-S. Review of second-order models for adsorption systems. *J. Hazard. Mater.* **136**, 681-689 (2006).
3. Sparks, D. Kinetics of Soil Chemical Processes Academic Press. *New York*, 119 (1989).
4. Weber, W. J. & Morris, J. C. Kinetics of adsorption on carbon from solution. *J. Sanit. Eng. Div.* **89**, 31-60 (1963).
5. Boyd, G., Adamson, A. W. & Myers, Jr. L. S. The exchange adsorption of ions from aqueous solutions by organic zeolites. II. Kinetics. *J. Am. Chem. Soc.* **69**, 2836-2848 (1947).
6. Tütem, E., Reşat, Apak., & Çağatay, F. Ü. Adsorptive removal of chlorophenols from water by bituminous shale. *Water Res.* **32**, 2315-2324 (1998).
